# Factors Influencing Pre-service Teachers' Intention to Use Digital Learning Materials: A Study Conducted During the COVID-19 Pandemic in Germany

**DOI:** 10.3389/fpsyg.2021.733830

**Published:** 2021-11-02

**Authors:** Jennifer Paetsch, Barbara Drechsel

**Affiliations:** ^1^Institute for Educational Science, University of Bamberg, Bamberg, Germany; ^2^Institute of Psychology, University of Bamberg, Bamberg, Germany

**Keywords:** COVID-19, self-regulated learning, resource-management strategies, teacher education, pre-service teacher, ICT in education, emergency remote teaching, higher education

## Abstract

The COVID-19 pandemic necessitated an abrupt change in university teacher education, in that most face-to-face courses were replaced by online education, which had a profound impact on students. Pandemic distance learning required students to possess a high degree of self-regulation concerning their learning environment and to find new ways of communicating with their peers and instructors. At the same time, the novel situation offered opportunities to experience new educational applications. To learn more about the possible benefits of distance learning, this study examines how the first online semester during the pandemic contributed to pre-service teachers' intentions to use digital learning materials in the future. Pre-service teachers enrolled in a German university (*n* = 348) answered an online questionnaire at the end of the summer term of 2020. Findings from structural equation modeling showed that the perceived quality of teacher training during the online semester and self-reported improvements in digital skills predicted significantly students' intentions to use digital learning materials for future teaching. Moreover, results revealed that attentional regulation predicted perceived quality of teacher training and self-reported improvements in digital skills during distance learning. Thus, attentional regulation had a significant indirect effect on pre-service teachers' behavioral intentions. The indirect effects of other resource management strategies (effort and time management) and intrinsic motivation were not significant. Our results show that the quality of online instruction was an important factor in student teachers' learning processes during the pandemic. Based on our results, we discuss implications for the promotion of pre-service teachers' intentions to use digital learning materials for teaching in schools.

## Introduction

The COVID-19 pandemic has necessitated a range of measures to restrict social contact. One of these has been the widespread introduction of distance learning in schools, colleges, and universities (UNICEF, 2020). In higher education (HE), this move has had a profound impact, with most face-to-face courses replaced by online education and only laboratory work in subjects such as medicine and chemistry remaining unaffected (Crawford et al., 2020). The extent to which HE has been digitalized was revealed by the first IAU—COVID-19 Global Impact Survey, which found that 85% of European institutions had replaced classroom teaching with remote instruction, while 15% had suspended or canceled teaching activities altogether (Marinoni et al., 2020). Overall, an extensive increase in digitalization in HE has occurred as a result of the pandemic.

The rapid transformation of teaching and learning settings (Crawford et al., 2020) has posed tremendous challenges across the education sector. Besides the administrative difficulty of procuring and installing appropriate technology, this “emergency remote teaching” (Hodges et al., 2020) has required university educators to manage the switch from traditional, in-class settings to various forms of distance education to ensure the continuation of lessons. At the same time, reduced access to the support systems and fixed structures that typify campus instruction has demanded higher levels of autonomy, self-regulation, and intrinsic motivation from students (Naujoks et al., 2021; Pelikan et al., 2021).

Although the switch to online teaching due to COVID-19 seemed sudden, a broader, technology-driven transformation of the educational landscape had long been underway. Innovations in information and communication technology (ICT) have led to the development of various applications to support learning processes (Koong and Wu, 2011; Hwang et al., 2015) by offering an active learning environment to students (de Koning-Veenstra et al., 2014). The potential benefits of ICT in learning and teaching at schools have received significant research attention and the results suggest that technology integration in the classroom is an important factor in enhancing learning processes (Chauhan, 2017; Zhu and Urhahne, 2018). Thus, ICT and digital instruction skills are viewed as crucial competencies for students starting into a professional career as teachers (Koehler and Mishra, 2009; Tondeur et al., 2012; Martin, 2015). Eventually, in-service teachers must be prepared and willing to support students' learning through the use of technology (Hatlevik and Hatlevik, 2018).

German teacher education^1^, like those of other countries, places great emphasis on developing special teacher training programs in the field of ICT. Specifically, pre-service teachers should develop their ability to apply technologies to pedagogical concepts and teaching practice (Mishra and Koehler, 2006). In turn, teacher educators are expected to equip pre-service teachers with the skills and motivation to use ICT for teaching and learning (Joo et al., 2018). From this perspective, the recent ubiquity of emergency remote teaching has provided both teacher educators and their students with the opportunity to expand their range of digital skills.

To explore the possible benefits of the pandemic-driven growth of distance learning in HE, this study examines how pre-service teachers' experiences during the initial distance learning semester (summer term, 2020) affected their intentions to use digital learning materials in future teaching. More specifically, the study investigated the relationships between pre-service teachers' intentions to use digital learning materials in their teaching, the quality of teacher training during the online semester, and self-reported changes in digital skills. Additionally, the role of personal resources such as internal learning strategies (namely attention, effort, and time management) and intrinsic motivation was explored.

### Digital Learning Environments in Initial Teacher Education

In recent years, emerging educational technology, such as web-based applications and collaborative tools, has expanded the available options for online learning in HE (e.g., Wong et al., 2019). Previous studies have demonstrated that instruction that is delivered entirely online is as effective as face-to-face instruction (Means et al., 2013). Although universities in many countries have developed teacher training programs for online instruction, digital learning environments are yet to be fully integrated into curricula. For example, a recent survey of higher education in Germany found that just 1.7% of universities rated the digitalization of teaching and learning in their institution as “well-advanced” (Gilch et al., 2019). Moreover, before COVID-19, the amount of teaching which could be conducted online was restricted by law at most German universities (Faller, 2015). Thus, when lockdown began, many initial teacher education programs were not fully prepared for online instruction by the start of the summer 2020 term (Zawacki-Richter, 2020). In contrast to planned and well-designed online learning environments, emergency remote teaching during COVID-19 has been characterized by a fast, temporary shift of instruction to an alternate delivery mode (Hodges et al., 2020). This mode was viewed as a specific form of online instruction in which neither teacher educators nor student teachers participated voluntarily (Hodges et al., 2020; Naujoks et al., 2021).

Initially, the pandemic meant that the homes of pre-service teachers were transformed into learning spaces consisting of asynchronous or synchronous online courses. The nature of this new digital learning environment has obstructed the learning process in various ways and thus has affected students' learning experiences. For students, the lack of in-class settings and fewer direct interactions may have required greater self-regulation and self-motivation, with reduced levels of support (Littlejohn et al., 2016; Naujoks et al., 2021). Pre-service teachers were required to plan, monitor, and control their learning processes more autonomously in order to follow self-study materials, organize participation in asynchronous and synchronous events, and communicate with peers and lecturers (Naujoks et al., 2021). Thus, the use of adequate self-regulated learning (SRL) strategies can be considered essential to the academic success of such students (Zimmerman, 2002; Naujoks et al., 2021).

Self-regulated learning has three key categories of learning strategies: cognitive, metacognitive, and resource management (Dresel et al., 2015; Panadero, 2017). Cognitive and metacognitive strategies are important for information processing and monitoring and verifying one's learning outcomes. Resource management is divided into external strategies (e.g., seeking help) or internal strategies, such as regulation of effort and attention, time management, and motivation (Dresel et al., 2015). SRL is crucial in learning environments that provide low levels of support and guidance (Wong et al., 2019) and in distance learning in particular (Zawacki-Richter, 2020; Naujoks et al., 2021). Prior studies have demonstrated that SRL strategies are positively correlated with academic success in online learning environments that afford high levels of learner autonomy (Broadbent and Poon, 2015; Broadbent, 2017). Specifically, internal resource-management strategies have proven to play an important role to achieve learning objectives in online learning (Broadbent and Poon, 2015; Broadbent, 2017; Kizilcec et al., 2017). *Thus, in situations where remote* learning, obligatory physical distancing, and a range of online platforms are widespread, internal resource-management strategies may be key to successful autonomous learning characterized by marked reductions in social support (Biwer et al., 2021).

Current empirical studies support this assumption. Pelikan et al. (2021) examined how students coped with the challenges of distance learning during the pandemic and found that students with high self-perceived competence reported higher levels of intrinsic motivation and elaborate learning strategies. However, the students in this study also noted significant obstacles to organizing their learning, keeping track of tasks, managing their time, and adhering to deadlines (Pelikan et al., 2021). Similarly, Biwer et al. (2021) investigated university students' adaption to emergency remote learning during the pandemic, with particular attention to resource-management strategies. Their findings indicate that students experienced greater difficulties in time management and regulating their attention and efforts. In addition, participants reported being less motivated by online than face-to-face education and also rated their general educational experience lower (Biwer et al., 2021). Finally, Naujoks et al. (2021) investigated students' use of external resource management strategies (e.g., environment structuring, time management, and help-seeking) during emergency remote teaching and differences between students' intended and actual use of them. They found that HE students were digitally prepared for online learning (e.g., they had access to necessary hardware and applications), but had not applied as many resource regulation strategies as intended before entering the remote learning environment.

The findings summarized above indicate that students are likely to encounter significant obstacles to their learning as a result of the switch to online instruction. At the same time, the lockdown provided a novel opportunity for pre-service teachers to improve their use of educational technology. However, two questions remain unanswered. First, has the digital competence of pre-service teachers increased as a result of the lockdown, and second, how has the digital learning experience contributed to pre-service teachers' intentions to use ICT materials in their professional lives?

### The Intention to Use ICT for Teaching and Learning

The policy impetus to foster teachers' use of ICT in teaching in school is grounded in (a) enhancing teaching and learning processes via digital media and (b) enabling students to participate fully in 21st-century societies by improving their digital literacy (OECD, 2015). The use of technology for educational purposes affords multiple opportunities to improve both teaching quality and learning outcomes (Koong and Wu, 2011; Hwang et al., 2015). Existing empirical studies indicate that the comprehensive embedding of technology into lessons can foster learning processes (Chauhan, 2017; Zhu and Urhahne, 2018). Integrating technology in this way works well with a variety of subjects, application types, and learning environments (Chauhan, 2017).

Although today's pre-service teachers commonly use ICT applications in their daily lives, the use of such apps for teaching and learning purposes is more problematic (Lei, 2009; Valtonen et al., 2011; Sailer et al., 2021). One possible reason is that pre-service teachers themselves have limited personal experience of digital learning environments (Lei, 2009; Valtonen et al., 2015). Despite their familiarity with various ICT applications, pre-service teachers show limited skills in utilizing these in teaching and learning (Lei, 2009; Valtonen et al., 2011), highlighting the need for initial teacher education programs to address the current deficit (Koehler and Mishra, 2009; Tondeur et al., 2012; Martin, 2015). Of particular importance is pre-service teachers' motivation to integrate technology into classroom practice (Backfisch et al., 2021a,b).

Thus, initial teacher education has the dual role of (a) providing pre-service teachers with opportunities to use digital learning materials and (b) motivating them to use ICT for teaching and learning in their professional lives. Indeed, teacher educators can serve as motivating models of good practice by using digital learning environments effectively themselves (Valtonen et al., 2015). The intention to use technology is defined “as the degree to which the user would like to use technology in the future” (Joo et al., 2018; p. 51) and it is assumed that the intention to use ICT in teaching is closely related to the user's acceptance of technology. In recent years, researchers have presented and tested several models to explain and predict the acceptance and use of IT among users (e.g., Wong, 2016). For instance, the technology-acceptance model (TAM) describes factors influencing teachers' acceptance and use of technologies (Teo, 2011; Wong, 2016; Scherer and Teo, 2019).

A recent meta-analysis of 45 studies deploying the TAM as a theoretical framework demonstrated that the intention to use ICT for teaching increases when teachers find educational technology both easy to use and useful (Scherer and Teo, 2019). Moreover, the analysis found that higher behavioral intentions were associated with higher degrees of technology integration (Scherer and Teo, 2019). Research also indicates that pre-service teachers' perceived self-efficacy, as well as the perceived ease of application and usefulness of technology, had a positive influence on their intention to use ICT in their future careers (Teo and Tan, 2012; Joo et al., 2018).

Thus, meaningful learning experiences with educational technology appear to be key to developing strong intentions to apply educational technology to teaching (Joo et al., 2018). Valtonen et al. (2015) showed that authentic learning experiences with ICT affected pre-service teachers' self-efficacy and subjective norms regarding technology, thus tilting them toward its use in teaching and learning. In the context of COVID-19, these findings prompt questions of whether and how the acceptance and use of technologies by pre-and in-service teachers depends on their experiences. In line with theoretical models, König et al. (2020) found that teachers' current ICT skills and opportunities to improve them were significant factors in their adoption of online teaching during school closures in Germany. Moreover, one recent qualitative study reported that online teaching during the pandemic led to a transition in teachers' identity and positively impacted their beliefs about ICT (Nazari and Seyri, 2021).

### Research Questions

The primary aim of this study, conducted during the pandemic, was to investigate the factors involved in pre-service teachers' intentions to use digital learning materials in their professional lives. The study first investigated the relationships between pre-service teachers' experiences of distance education during the first online semester and pre-service teachers' intentions to use digital learning materials for teaching. It was hypothesized that the perceived quality of university teacher training (*hypothesis 1*) and self-reported improvements in digital skills during emergency remote teaching (*hypothesis 2*) will predict the intention to use digital learning materials for teaching in the future.

**Figure 1:**
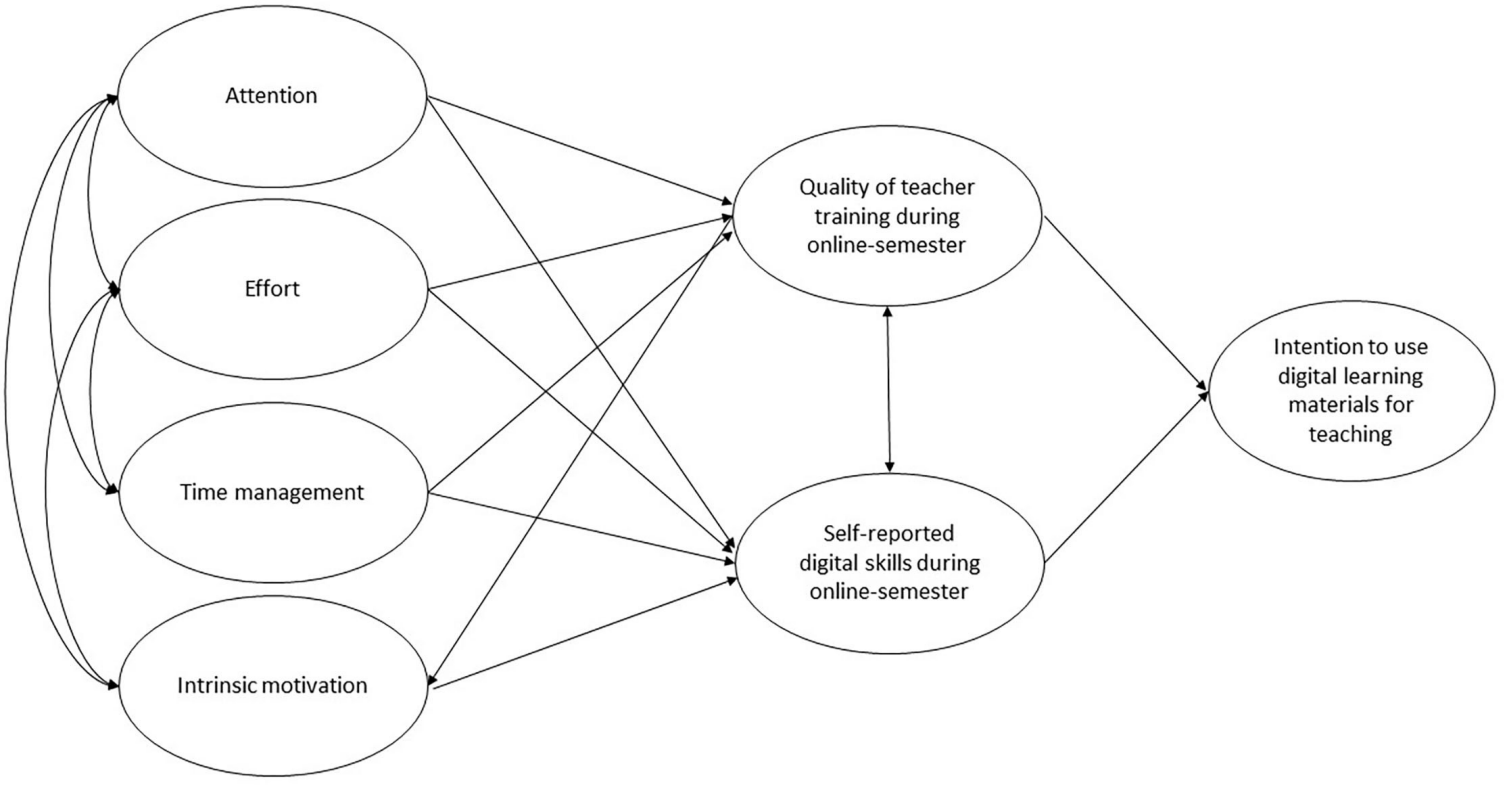
Hypothetical model.

The second area of investigation was the role of internal resource management strategies and intrinsic motivation in this context. We hypothesized that intrinsic motivation, effort regulation, time management, and attentional regulation are associated with perceived quality of teacher training (*hypothesis 3*) and self-reported enhancement of digital skills during distance learning (*hypothesis 4*). Thus, intrinsic motivation and resource management strategies will have an indirect effect on pre-service teachers' intention to use digital learning materials for teaching (*hypothesis 5*). The hypothetical model is displayed in Figure 1.

## Materials and Methods

### Sample

A total of 348 pre-service teachers (84.4% female, 17.7% male, and 0.9% non-binary) studying different combinations of subjects participated in the research study. All students were enrolled at the University of Bamberg and aimed to teach at a range of school types. Among the participants, 46.6% intended to work in elementary school, 13.5% at secondary school/middle school, 23.9% at high school/gymnasium, and 16.1% at vocational schools. The mean age of the participants was 22.5 years (SD = 3.1) with a mean study duration of 5.1 semesters (SD = 2.8).

Participants were asked about their online activities during the first distance learning semester using the following question: *How often have you participated in synchronous lectures (real-time teaching, i.e., Zoom) and asynchronous lectures (not limited to a specific point of time, i.e., video or audio recording) during the semester?* The findings showed that 82.5% of the students had attended at least eight synchronous lectures and 68.9% of the students had been present at least eight asynchronous lectures. In addition, students were asked about their workloads: most students (79.9%) reported their online classroom hours as over 10 hours per week and 81.3% of students claimed that their general workload (including self-study) was higher than in the previous (regular) semester.

### Procedures

Data were collected through an online survey administered at the end of the first online semester, in July 2020. The university had undertaken to provide online education throughout the semester, with educators designing and organizing their courses autonomously. Our research study was announced and the survey was distributed via websites, e-mail, and social media. Pre-service teachers completed an online questionnaire. The participation of all student teachers was voluntary, and they were informed at a preliminary stage about the objectives of the investigation and how the data would be used in keeping with the ethical guidelines of human subject research. The confidentiality of the data and anonymity of participants were also assured.

### Measures

The novel scales developed for this research reflected the circumstances of the pandemic. To ensure the construct validity of the measures, confirmatory factor analyses (CFA) were conducted.

#### Intention to Use Digital Learning Materials

To assess the subjects' intention to use digital learning materials we newly designed a scale consisting of two items, as follows: 1. *I will use some of the computer programs that I worked with in the context of digital teaching for my future teaching profession*. 2. *The experience I have gained in the context of digital teaching proves useful for my future teaching profession*. Respondents were asked to indicate the extent to which they agree to the items on a 7-point Likert-type scale (1 = very strongly disagree to 7 = very strongly agree). The reliability coefficient of the scale is α = 0.81.

#### Quality of Teacher Training During the Online Semester

The second scale consisted of six items to measure students' perspectives on the quality of teacher training during the online semester. The time before distance learning was used as a benchmark in questions such as the following: *Compared to the time before the COVID-19 outbreak, how do you rate your experience of the average quality of instruction?* Respondents were asked to indicate the extent of their agreement with each item on a 5-point Likert-type scale (1 = much worse to 5 = much better). The reliability coefficient of the scale is α = 0.85.

#### Self-reported Changes in Digital Skills

This scale comprised four items assessing students' self-reported digital skills during the online semester with the time before the semester as the benchmark from which changes in self-assessed digital skills were measured. A sample item on the scale is as follows: *Compared to the time before the COVID-19 outbreak, how do you rate your digital expertise?* Respondents indicated the extent of their agreement on a 5-point Likert-type scale (1 = much worse to 5 = much better). The reliability coefficient of the scale is α = 0.89.

#### Intrinsic Motivation

The 3-item measurement of motivational regulation for learning in university students scale (SMR-LS) was developed by Thomas et al. (2018). It was based on Deci and Ryan's Self-Determination Theory (Deci and Ryan, 2002). We used the scale in the current study to measure pre-service teachers' intrinsic motivation. A sample item on the scale is as follows: *Currently, I enjoy studying*. Respondents were asked to indicate the extent to which they agreed to the items on a 7-point Likert-type scale (1 = very strongly disagree to 7 = very strongly agree). The reliability coefficient of the scale is α = 0.90.

#### Strategies for Managing Internal Resources

The use of internal regulation strategies (attention, effort, and time management) was assessed with three scales from Klingsieck (2018) learning strategies of university students (LIST-K). All items were based on a five-point scale ranging from 1 (rarely) to 5 (very often). Attention (α = 0.89) was assessed by three items. A sample item on the scale is as follows: *While studying I'm easily distracted*. Effort (α = 0.62) was assessed by two items. A sample item on the scale is as follows: *I don't give up, even if the content is difficult and complex*. Time management (α = 0.81) was assessed by two items. A sample item on the scale is as follows: *While studying I stick to a specific timetable*.

### Statistical Analysis

Data analysis was performed using IBM SPSS Statistics 26.0 (IBM Corp, 2017) and Mplus 7.4 (Muthén and Muthén, 2012). The percentage of missing values at the item level was low (max 5.46%). To deal with the small number of missing values, the full information maximum likelihood approach (FIML) implemented in Mplus was employed. Robust maximum likelihood (MLR) estimation was most appropriate to the Likert scales employed in the items. Significance testing was performed at the 0.05 level.

Confirmatory factor analyses were conducted to analyze construct validity, with two CFA models constructed for the seven constructs. The indicators of the latent variables were the items of the different scales. Structural equation modeling (SEM) was used to analyze the relationships of the hypothetical model. SEM is a multivariate quantitative technique used to estimate the relationships among observed variables to validate a theoretical model (Thakkar, 2020). The effects of the study duration and the school type (elementary school vs. other school types) were controlled for. Additionally, indirect effects on the intention to use digital learning materials were investigated by decomposing the total effect into a set of direct and indirect effects.

Several indices were used to evaluate the model. We deployed the χ^2^/df test (<5), the root mean square error of approximation (RMSEA), the comparative fit index (CFI), the Tucker–Lewis index (TLI), and the standardized root mean square residual (SRMR). We utilized widely-used cutoff scores reflecting excellent and adequate fit to the data: TLI and CFI values above 0.95 or 0.90; RMSEA values below 0.06 or 0.08; and SRMR values below 0.08 (Hu and Bentler, 1999).

## Results

### Descriptive Results and Construct Validity of Scales

The descriptive results, correlations, and reliability scores of the constructs are presented in Table 1. There were significant correlations among all variables, except for time management, which only correlated significantly with the two other internal regulation strategies and self-reported skills. Small to medium effect sizes were found for all other correlations. The mean scores for intention to use digital learning materials (*M* = 4.53, *SD* = 1.57), self-reported digital skills (*M* = 3.73, *SD* = 0.63), intrinsic motivation (*M* = 4.61, *SD* = 1.31) and effort (*M* = 3.92, *SD* = 0.76) exceeded the midpoint of a 5-point (3) or 7-point scale (4), indicating that students had assessed themselves as strong in these areas. The mean scores for quality of teacher training (*M* = 2.85, *SD* = 0.70), attention (*M* = 2.58, *SD* = 1.02), and time management (*M* = 2.77, *SD* = 1.09), however, were just below the midpoint, indicating less confidence in these areas.

**Table 1:** Descriptive results, correlations, and reliabilities.

	**1**	**2**	**3**	**4**	**5**	**6**	**7**
1. Intention to use digital learning materials							
2. Quality of teacher training	0.38^**^						
3. Self-reported digital skills	0.23^**^	0.40^**^					
4. Intrinsic motivation	0.22^**^	0.25^**^	0.22^**^				
5. Internal regulation strategies: attention	0.25^**^	0.41^**^	0.33^**^	0.24^**^			
6. Internal regulation strategies: effort	0.14^**^	0.15^**^	0.18^**^	0.15^*^	0.35^**^		
7. Internal regulation strategies: time management	0.02	0.09	0.13^*^	0.09	0.34^**^	0.32^**^	
Means	4.53	2.85	3.73	4.61	2.58	3.92	2.77
SD	1.57	0.70	0.63	1.31	1.02	0.76	1.09
Min	1	1.17	1	1	1	1	1
Max	7	5	5	7	5	5	5
Cronbach's alpha	0.81	0.85	0.89	0.90	0.89	0.62	0.81
*N*	344	316	329	340	341	341	339
Missing values	4	32	19	4	7	7	9

** *Correlation is significant at the 0.01 level (2-tailed)*,

* *Correlation is significant at the 0.05 level (2-tailed)*.

In addition, two separate CFAs were conducted to confirm the factor structures of the latent variables. The first 3-factor CFA model included a total of 12 items measuring pre-service teachers' intentions to use digital learning materials, the quality of teacher training during distance learning, and self-reported digital skills. The indices indicated good data fit (χ^2^ = 107.39, *df* = 51, *p* < 0.001, RMSEA = 0.06, SRMR = 0.04, TLI = 0.96, and CFI = 0.97) with factor loadings ranging from 0.60 to 0.92. The second 4-factor CFA model included 11 items measuring intrinsic motivation and the internal regulation strategies of attention, effort, and time management. The indices for this model again indicated a good fit to the data (χ^2^ = 78.73, *df* = 38, *p* < 0.001, RMSEA = 0.06, SRMR = 0.04, TLI = 0.96, and CFI = 0.97) with factor loadings ranging from 0.62 to 0.93. The factor loadings for both CFA models are reported in Table 2. These results indicated that the construct validity of all of the scales was acceptable, and all of the latent variables were well-represented by the indicators.

**Table 2:** Standardized factor loadings for the items in the CFA models.

**CFA models**	**Latent variable**	**Item**	**Factor loadings**
Model 1 Intentions to use digital learning materials, quality of teacher training, and self-reported digital skills.	Intentions to use digital learning materials	1	0.74
		2	0.92
	Quality of teacher training	1	0.79
		2	0.65
		3	0.79
		4	0.65
		5	0.60
		6	0.77
	Self-reported digital skills	1	0.81
		2	0.85
		3	0.77
		4	0.81
Model 2 Intrinsic motivation and internal regulation strategies	Intrinsic motivation	1	0.86
		2	0.90
		3	0.82
	Attention	1	0.84
		2	0.93
		3	0.81
	Effort	1	0.73
		2	0.62
	Time management	1	0.76
		2	0.84
		3	0.70

### Results of the Structural Equation Modeling

The structural model was tested to examine the direct and indirect relationships between the intention to use digital learning materials for teaching, the quality of teacher training during distance learning, self-reported digital skills during distance learning, intrinsic motivation, and internal regulation strategies (attention, effort, and time management). The indices indicated an excellent fit for the model (χ^2^ = 348.92, *df* = 241, χ^2^/*df* = 1.45, RMSEA = 0.04 [0.027, 0.044], SRMR = 0.04, TLI = 0.96, and CFI = 0.97).

**Figure 2:**
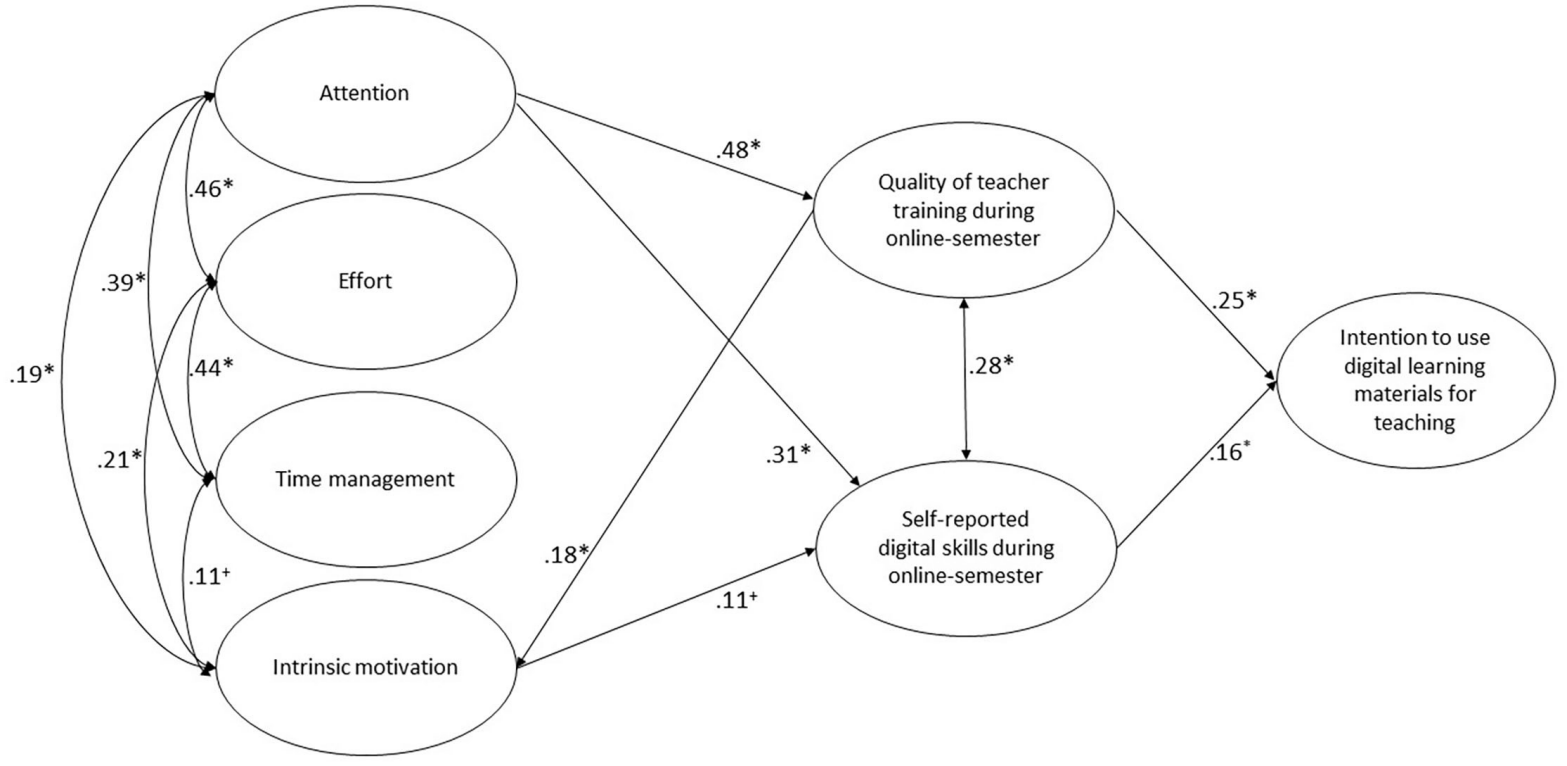
Structural equation model. Only paths *p* < 0.10 are displayed. *p < 0.05; ^+^*p* < 0.10. The included control variables *study duration* and *school type* are not illustrated. See **Table 3** for the effects. See **Table 4** for direct and indirect effects among variables.

These findings reveal that the perceived quality of teacher training during the online semester (β = 0.25, *p* < 0.05) and self-reported digital skills (β = 0.16, *p* < 0.05) were significant predictors of students' intention to use digital learning materials for teaching (see Figure 2). These results thus supported hypothesis 1 and hypothesis 2.

The internal regulation strategies of attention, effort, and time management had no direct effects on students' intention to use digital learning materials for teaching. However, attention was a significant predictor of the perceived quality of teacher training during the online semester (*hypothesis 3*, β = 0.48, *p* < 0.05) and self-reported digital skills (*hypothesis 4*, β = 0.31, *p* < 0.05). The internal strategies of effort and time management did not predict student perceptions of training quality. Thus, hypotheses 3 and 4 can only be confirmed for the internal regulation strategy of attention.

There was a positive correlation between the perceived quality of teacher training and self-reported digital skills (β = 0.28, *p* < 0.05). Intrinsic motivation was predicted by the perceived quality of teacher training during the online semester (β = 0.18, *p* < 0.05) and positively correlated with attention (β = 0.19, *p* < 0.05), and effort (β = 0.21, *p* < 0.05). For the control variable of study duration, only significant effects on time management (β = 0.12, *p* < 0.05) and attention (β = 0.12, *p* < 0.05) were detectable (see Table 3). For the control variable of school type (elementary school vs. other school types) significant negative effects on students' intention to use digital learning materials for teaching (β = −0.24, *p* < 0.05), intrinsic motivation (β = −0.11, *p* < 0.05), and attention (β = −0.12, *p* < 0.05) were observable (see Table 3). This means that students, who intended to work in elementary school showed, on average, significantly less values on this scales than students, who intended to work in other school types.

**Table 3:** Standardized effects for the included control variables *study duration* and *school type* (elementary school vs. other).

**Variables**	**Study duration**		**Elementary school**	
	**β**	***p***	**β**	***p***
Intention to use digital learning materials for teaching	−0.06	0.36	−0.24^*^	< 0.01
Self-reported digital skills	0.03	0.53	0.02	0.78
Quality of teacher training	−0.09	0.10	−0.03	0.58
Attention	0.12^*^	0.04	−0.12^*^	0.04
Effort	0.12	0.07	−0.01	0.85
Time management	0.12^*^	0.04	0.09	0.14
Intrinsic motivation	−0.01	0.87	−0.11^*^	0.05

* *Significant at the 0.05 level (2-tailed)*.

### Indirect Effects on Students' Intention to Use Digital Learning Materials for Teaching

Direct and indirect effects on students' intention to use digital learning materials for teaching were estimated in Mplus using model indirect. As shown in Table 4, among all predictors, only attention (*z* = 0.18, *p* < 0.01) had a significant indirect effect on the intention to use digital learning materials for teaching (*hypothesis 5*). The direct relationship between attention, effort, time management, intrinsic motivation, and intention to use digital learning materials for teaching was not statistically significant (see Table 4). Thus, hypothesis 5 is supported only for attention.

**Table 4:** Standardized indirect effects on intention to use digital learning materials for teaching.

**Predictors**	**Standardized estimates of direct effect**	**Standardized estimates of indirect effect**	**Standardized estimates of total effect**
Attention	0.04	0.18^*^	0.22^*^
Effort	0.11	0.02	0.12
Time management	−0.07	−0.02	−0.10
Intrinsic motivation	0.08	0.02	0.10

* *Significant at the 0.05 level (2-tailed)*.

## Discussion

Against the backdrop of the switch to distance education necessitated by the COVID-19 pandemic, the present study aimed to shed light on pre-service teachers' experiences during the challenging situation and to identify key factors in students' intentions to use digital learning materials in their future teaching profession. We argue that under the conditions of emergency remote teaching, with traditional learning formats transformed into online provision, pre-service teachers' experience with technology has increased the likelihood that they will use it in their future careers. In Germany, digital learning had not been fully integrated into HE before the pandemic struck (Gilch et al., 2019). Therefore, the rapid shift to online education has enabled students to gain a deeper understanding of how technology might be integrated into teaching and the benefits this may confer (Lei, 2009; Valtonen et al., 2015).

First, the study investigated the relationship between pre-service teachers' intentions to use digital learning materials in their teaching and (a) the perceived quality of teacher training and (b) self-reported improvements in digital skills at a large public university. Our descriptive findings indicate that, despite the sudden shift from the traditional, classroom-based education format to a remote format, pre-service teachers evaluated the quality of initial teacher training during the first online semester as equivalent, on average, to the quality of traditional, pre-pandemic initial teacher training. Furthermore, on average, students reported improvements in their digital skills. As expected, our results demonstrate that the perceived quality of teacher training during COVID-19 had a notable and significant impact on students' intentions to use digital learning material in their future work as teachers. Pre-service teachers who perceived the quality of emergency remote teaching to be equivalent to or better than their pre-COVID experiences reported stronger intentions to use ICT in their work. The analyses results support those of previous studies that identified meaningful learning experiences with educational technology as a key factor in the development of such intentions (Valtonen et al., 2015).

Additionally, self-reported digital skills proved to have a statistically significant influence on students' intentions. This finding is in line with other studies whose results suggested that pre-service teachers' self-efficacy and skills predict behavioral intentions (Valtonen et al., 2015; Joo et al., 2018). However, the effect is very small what may be due to our focus on the improvement of students' digital skills and thus on the effect of online learning in the pandemic. Specifically, pre-service teachers' self-reported digital skills were compared with a pre-pandemic baseline, meaning that some students may have felt they had already acquired skills which the online semester had not necessarily improved. The assessment of teacher training quality, however, was positively correlated with changes in self-reported digital skills. Thus, pre-service teachers who perceived that the quality of emergency remote teaching exceeded or equaled the traditional instruction they had experienced before the outbreak of COVID-19 reported larger gains in digital skills. From a theoretical perspective, it seems reasonable to suggest that the relationship would be bidirectional: on the one hand, improved online instruction might help to develop students' ICT skills, while on the other hand, improved digital skills might allow for more participation and investment in online courses, thus influencing students' perceptions of quality.

Effects of different school types of pre-service teachers were also analyzed. As elementary school teachers will teach students in a different age group than secondary school teachers, the use of digital applications in the classroom might be different for different school types. Furthermore, there are differences between students' entry characteristics concerning school type (Retelsdorf and Möller, 2012). The results reveal that students who intended to work in elementary school showed, on average, significantly fewer intentions to use digital learning materials in their future work as teachers. This finding might be explained by the particularities of ICT integration into classroom environment for young children. This result highlights the need to differentiate between pre-service teachers with different study objectives.

Another area of investigation focused on the relationship between internal resource management strategies (attention, effort, and time management), intrinsic motivation, and instructional quality, as well as skill improvement. Previous studies have shown that all these are important factors in online learning environments characterized by high levels of learner autonomy (Broadbent and Poon, 2015; Broadbent, 2017; Kizilcec et al., 2017). However, the regulative resource of attention was found to be the only significant factor in participants' perception of instructional quality and skills. As anticipated, pre-service teacher who reported higher levels of attention regulation also perceived the quality of teacher training to be higher and reported greater gains in self-reported digital skills. This corroborates the findings of Biwer et al. (2021) who detected a positive correlation between attention and the educational experience of HE students during the pandemic.

Contrary to expectations, neither pre-service teachers' regulation of effort nor time management strategies predicted their perceived quality of teacher training or self-reported digital skills. This is surprising as an increased need for self and time management was reported by Reinhold et al. (2021) following the recent switch to distance learning. Biwer et al. (2021) also found positive associations between effort, time management, and educational experience during the crisis. However, the authors only reported bivariate relationships between these factors, whereas our use of structural equation modeling enabled complex relationships to be detected. The findings of the current study may be explained by the fact that demands on students' time management were not particularly severe during this phase of the pandemic since social distancing rules severely restricted leisure opportunities of any sort, thus freeing up additional time for study. This assumption aligns with the finding of Naujoks et al. (2021) that time management strategies were less often used during the online semester than students had previously intended. Similarly, Zhang et al. (2021) indicated that students succeeded in completing their assignments in the first online semester.

As expected, students' perception of instructional quality had a significant impact on intrinsic learning motivation. Pre-service teachers who perceived the quality of emergency remote teaching to be equivalent to or better than their pre-COVID experiences reported greater intrinsic motivation for learning. This result aligns with the finding of Biwer et al. (2021) that students' motivation correlated with educational experience during the pandemic. Although intrinsic learning motivation in our study did not make a significant contribution to digital skill improvement, results from Pelikan et al. (2021) indicate that students with high perceived competence have higher intrinsic motivation than students with low perceived competence during distance learning. As mentioned above, we measured only perceived digital skill improvement, thus pre-service teachers' general perceived competence was not taken into account.

The last area of investigation focused on indirect effects on intention to use digital learning materials for teaching. Among the intrinsic motivation and resource management strategies investigated in this study, only the regulation of attention was found to indirectly affect pre-service teachers' intention to use digital learning materials for teaching. There were no significant contributions of effort and time management to participants' intention to use digital learning materials for teaching. However, there are substantial relationships between the three internal resource management strategies. Hence, the findings further confirm both the importance of internal resource management to successful online learning during the pandemic and to pre-service teachers' willingness to integrate technology into their future teaching profession. The results indicate that students who positively evaluate their experience of distance learning, which is linked to their capacity to regulate their attention, might be more willing to integrate technology in their classroom in their future careers. Overall, the current study contributes to the literature by underlining the importance of well-designed digital learning environments in initial teacher education. It also highlights the positive effects of digitalization in teacher education conferred by the switch to remote distance learning during the pandemic.

### Limitations and Future Directions

The first limitation of this study is that the sample consisted of volunteer subjects and therefore was not representative of the population of pre-service teachers. Given that self-regulatory resources were a key area of investigation, the voluntary basis of participation may have skewed the sample toward students with a higher capacity for self-regulation: participants with less of a capacity for self-regulation during the pandemic may have felt unable to take or complete the survey, or may have missed the lectures in which the study was announced. This students may have been unintentionally excluded from our sample, thus potentially biasing the results. However, the composition of the sample was manifold, with pre-service teachers of all school types and with different study durations (i.e., number of semesters student teachers completed in summer term 2020).

The second limitation of this study is that the validity of the newly designed instruments was not verified. Due to the novelty of the pandemic situation, it was necessary to develop items to suit the current circumstances. We measured the subjects' intention to use digital learning materials with a scale consisting of only two items, which may have affected its validity. However, the results of the CFA confirmed the factor structures of the latent variables. Further research is needed to verify the psychometric parameters of the reliability and validity of the instruments developed in our study.

A third limitation stems from the study's reliance on self-report measures, which can elicit socially desirable responses from participants and therefore lead to results that differ markedly from those obtained by other methods such as behavioral observation. Pre-Service teachers' self-reports were compared with a pre-pandemic baseline, meaning that the general level of current digital skills and instructional quality was not taken into account. Various constraints meant that some factors were excluded from the model. For example, data on participants' achievements, individual use of distance learning, such as time spent on courses or number of interactions, general motivational profile, or their personal situation were not analyzed. Multiple additional factors influencing the intention to use digital learning materials remain uninvestigated (e.g., self-efficacy of using educational technologies), but these factors lay beyond the scope of the current study. Future research on the intentions to use digital learning materials for teaching could include more aspects of students' personal resources (e.g., self-efficacy), prior experiences with digital learning environments, and professional knowledge to generate deeper insights. Additionally, a longitudinal design would allow for insights into changes in intentions and provide further information on the underlying mechanisms that influence behavioral intentions concerning technology integration in education. Moreover, further research is needed to examine characteristics of digitalization in initial teacher education under non-pandemic circumstances and its impact on pre-service teachers' behavioral intentions concerning educational technology integration.

Nonetheless, these limitations of the study are in part counterbalanced by the provision of valuable information on the experiences of a group of students at a unique and highly challenging time and evidence supported recommendations for improving practice in teacher education.

### Implications for Initial Teacher Education and Conclusion

This study offers insights into how pre-service teachers experienced emergency remote teaching and how the sudden transformation of teacher training from a traditional classroom-based format into a digital format may have affected their intentions for future teaching. While universities will eventually switch back to face-to-face teacher training, online learning and technology integration are likely to remain part of initial teacher training. Online learning settings differ meaningfully from traditional higher education settings, in that the online learning settings require a greater degree of autonomous learning situations. Hence, fostering resource management strategies seems to be a promising approach.

In conclusion, this study provides robust evidence that university teaching matters to future teachers in terms of building intentions and shaping professional beliefs. It demonstrates the value of providing pre-service teachers with meaningful and adaptive opportunities to learn at university. In addition this study shows that effective teaching fosters the readiness and intention of students to deploy a range of ICT resources in their professional lives. As mentioned above, teacher training must confront a particular challenge within the process of digital transformation. Specifically, universities must attend carefully to their role as learning organizations since they are better placed to integrate ICT than institutions of primary and secondary education. Universities must provide pre-service teachers with state-of-the-art models of teaching so they can apply these models to their own professional lives. However, teacher educators are not a homogeneous group (Daumiller et al., 2021; Scherer et al., 2021) and the comprehensive changes to practice imposed by the pandemic have been implemented in a range of different ways. Nonetheless, the challenge of developing the knowledge, competence, and motivation of teacher educators concerning digital teaching must remain a key goal of teacher training education.

## Data Availability Statement

The datasets presented in this article are not readily available because informed consent signed by participants stated that data were only accessible to the authors of this study. Requests to access the datasets should be directed to Jennifer Paetsch, Jennifer.paetsch@uni-bamberg.de.

## Ethics Statement

Ethical review and approval was not required for the study on human participants in accordance with the local legislation and institutional requirements. The patients/participants provided their written informed consent to participate in this study.

## Author Contributions

JP performed the statistical analyses and drafted the manuscript. BD contributed to the conception of this study, discussed the results of the analyses, contributed to the manuscript, and the revisions. All listed authors read and approved the submitted manuscript.

## Funding

This project is part of the Qualitätsoffensive Lehrerbildung, a joint initiative of the Federal Government and the *Länder* which aims to improve the quality of teacher training. The program is funded by the Federal Ministry of Education and Research (01JA1915). The authors are responsible for the content of this publication.

## Conflict of Interest

The authors declare that the research was conducted in the absence of any commercial or financial relationships that could be construed as a potential conflict of interest.

## Publisher's Note

All claims expressed in this article are solely those of the authors and do not necessarily represent those of their affiliated organizations, or those of the publisher, the editors and the reviewers. Any product that may be evaluated in this article, or claim that may be made by its manufacturer, is not guaranteed or endorsed by the publisher.

## Footnotes

^1^In Germany, all teachers are educated and trained in a process consisting of two phases. During the first phase, pre-service teachers attend university and study two or three school subjects and their subject-didactics. In addition, they study educational topics and have several internships in schools.
